# A replication-competent foot-and-mouth disease virus expressing a luciferase reporter

**DOI:** 10.1016/j.jviromet.2017.05.011

**Published:** 2017-09

**Authors:** Fuquan Zhang, Eva Perez-Martin, Nick Juleff, Bryan Charleston, Julian Seago

**Affiliations:** The Pirbright Institute, Ash Road, Pirbright, Woking, Surrey GU24 0NF, UK

**Keywords:** FMDV, foot and-mouth disease virus, qRT-PCR, quantitative reverse transcription-PCR, RLU, relative light unit, hpi, hours post infection, pfu, plaque forming unit, MOI, multiplicity of infection, GFP, green fluorescent protein, Foot-and-mouth disease virus, Bioluminescence, Luciferase, Reverse genetics

## Abstract

Bioluminescence is a powerful tool in the study of viral infection both *in vivo* and *in vitro*. Foot-and-mouth disease virus (FMDV) has a small RNA genome with a limited tolerance to foreign RNA entities. There has been no success in making a reporter FMDV expressing a luciferase in infected cell culture supernatants. We report here for the first time a replication-competent FMDV encoding Nanoluciferase, named as Nano-FMDV. Nano-FMDV is genetically stable during serial passages in cells and exhibits growth kinetics and plaque morphology similar to its parental virus. There are applications for the use of Nano-FMDV such as real-time monitoring of FMDV replication *in vitro* and *in vivo.*

## Highlights

• We have generated a replication-competent foot-and-mouth disease virus expressing Nanoluciferase, designated as Nano-FMDV.
• Nano-FMDV is genetically stable.
• The replication of Nano-FMDV can be monitored by bioluminescent methods.
• This reporter virus has potential applications in real-time monitoring of FMDV infection *in vitro and in vivo*, and in screening of antivirals and antibodies.

## 1 Introduction

Foot-and-mouth disease virus (FMDV) causes foot-and-mouth disease (FMD) in cloven-hoofed farm animals (Grubman and Baxt, 2004). Viral particles have a positive-sense RNA genome of approximately 8500 nt consisting of a 5′ untranslated region (UTR), a single open reading frame (ORF), and a 3′ UTR with a polyadenylic acid [poly(A)] tail. The ORF encodes a polyprotein that is processed by a viral encoded protease into at least 13 proteins; four structural (VP1, VP2, VP3 and VP4) and nine non-structural proteins (leader proteinase (Lpro), 2A, 2B, 3A, 3B1, 3B2, 3B3, 3Cpro and 3Dpol). FMD outbreaks have devastating economic and social impacts (Knight-Jones and Rushton, 2013), however many aspects of the FMDV life cycle, cell tropism and pathogenesis are still not well characterised. There is a need to develop new tools such as a reporter virus that can be used to trace infection *in vitro* and *in vivo* in a real-time manner (Robinson et al., 2016).

FMDV has a diameter of 25 nm (Wild et al., 1969) and a small, highly structured RNA genome, which offers limited packaging capacity and choices of insertion sites for foreign reporter genes. Our early attempts to generate recombinant FMDV expressing green fluorescent protein (GFP) of *Aequorea victoria* or the *Renilla* luciferase protein (RL) of *Renilla reniformis*, which are about 720 nt and 930 nt respectively, were unsuccessful. A panel of truncated GFP gene fragments were subsequently used to show a packaging limit of between 417 nt and 504 nt for the targeted insertion site located between VP1 and 2A in the FMDV genome. Based upon this finding we successfully constructed a viable reporter FMDV expressing a fluorescence protein, termed iLOV (Seago et al., 2013, Chapman et al., 2008). Although iLOV-FMDV has particular application for monitoring FMDV infection in real-time with microscopy, emitted levels of fluorescence are relatively faint and an alternative FMDV reporter is required to develop quantitative assays (Seago et al., 2013).

Recently Promega has developed a new luminescent reporter, namely Nanoluciferase, NanoLuc^®^ (NLuc), which is smaller and brighter than other luciferases available (Hall et al., 2012a). This prompted us to construct a recombinant FMDV expressing NLuc so that we can make use of the powerful bioluminescence technique in various fields of FMDV study. We report here that we have rescued an infectious FMDV expressing NLuc and investigated its application to monitor infection *in vitro*.

## 2 Material and methods

### 2.1 Cell lines

Cells were maintained at 37 °C in 5% CO_2_. IB-RS2 cells were cultivated in Glasgow’s modified Eagle’s medium (GMEM, Sigma) with 10% foetal calf serum (FCS), BHK-21 cells in GMEM with 10% FCS and 5% tryptose phosphate broth, and ZZ-R 127 cells in Dulbecco’s modified Eagle’s medium/Ham’s F12 (Sigma) with 10% FCS. All media were supplemented with 100 SI units/ml penicillin and 100 μg/ml streptomycin (all from Sigma).

### 2.2 Construction of infectious copy plasmids

New recombinant viruses were generated from a previously described (Seago et al., 2012) infectious copy plasmid, herein termed AvrII-O1K/OUKG. In brief, AvrII-O1K/OUKG encodes the VP2, VP3, VP1 and the 2A proteins of FMDV O UKG/35/2001 and the Lpro, VP4, 2B, 2C, 3A, 3B, 3C and 3D proteins of FMDV O1K. The AvrII-O1K/OUKG genome contains a unique Avr II site between VP1 and 2A for insertion of exogenous cDNAs that encode reporter proteins (Botner et al., 2011). In this study, one targeted amino acid mutation (VP3 56 His to Arg) was introduced into the AvrII-O1K/OUKG infectious clone using the QuikChange Lightning Mutagenesis kit (Agilent Technologies), producing a new parental infectious copy plasmid termed AvrII-O1K/OUKG-_HS+_. This targeted mutation enabled the use of heparan sulphate for viral entry and thus virus infection of BHK-21 cells (Fry et al., 2005, Jackson et al., 1996). The Avr II sites of both AvrII-O1K/OUKG and AvrII-O1K/OUKG-_HS+_ were then used for insertion of DNA encoding the NanoLuc open reading frame, which was PCR amplified from plasmid pNL1.1 (Promega) using the following primer pair: forward/5′-*AGG*CTCTAGAATGGTCTTCACACTCGAAGA-3′ and reverse/5′-*ATA*CCTAGGCGCCAGAATGCGTTCGCAC-3′ (clamp bases in italics). Xba I (TCTAGA) and Avr II (CCTAGG) sites (underlined in primers) were engineered to the 5′ and 3′ ends of the amplicon to facilitate cloning and screening. The resultant infectious copy plasmids were termed Nano-FMDV and Nano-FMDV-_HS+_, respectively. Construction of infectious copy plasmids and all virus work was performed at a SAPO4 level of biocontainment.

### 2.3 *In vitro* RNA transcription and transfection

RNA was transcribed from infectious copy plasmids using the MEGAscript T7 kit (Ambion). After RNA synthesis was complete, the *in vitro* transcription reactions were treated with 1 μl of RNase-free DNase (Ambion) at 37 °C for 15 min to degrade the DNA templates and the RNA was purified using the MEGAclear kit (Ambion). The TransIT-mRNA Kit (Mirus Bio Corporation) was used to deliver infectious RNA into BHK-21 cells. After 24–48 h, the cells were freeze-thawed to release the initial virus stock, designated as passage 0 (P0). ZZ-R 127 cells (Brehm et al., 2009) were used for the subsequent passages to optimise the rescue of recombinant virus and maximise virus yields.

### 2.4 Luciferase assay

The Nano-Glo^®^ Luciferase Assay System (Promega) was used according to the manufacturer’s instructions. Briefly, 10 μl of infected supernatant was mixed with an equal volume of assay buffer containing the Nano-Glo substrate in a white 96-well plate (Nunc, Thermo Scientific) and incubated at room temperature for 10 min. The plate was then read in a Synergy 2 plate reader (BioTek) and light signal determined as Log_10_ relative light units (RLU)/10 μl.

### 2.5 Plaque assay

IB-RS2, BHK-21 or ZZ-R 127 cells were plated in 6 or 24-well plates to produce confluent cell monolayers the following day. Cells were infected by incubation with ten fold dilutions (made in 1 X PBS) of P3 virus stocks for 1 h at 37° C and then overlaid with medium containing 0.6% UltraPure LMP Agarose (Invitrogen). Cells were fixed and stained after 24 h with a solution containing 4% formaldehyde and crystal violet or methylene blue before removal of the overlay. Viral titres were determined as Log_10_ plaque forming units (PFU)/ml.

### 2.6 Virus growth curve

ZZ-R 127 cells grown in 6-well plates were infected with the respective FMDV stocks (P3) at a multiplicity of infection (MOI) of 0.001 by incubation for 1 h at 37° C. Inoculum was then discarded and the monolayers were washed with PBS and 2-morpholinoethanesulfonic acid (MES) buffered saline (25 mM MES [pH 5.5], 145 mM NaCl) to remove non-adsorbed virus and Nanoluciferase carried over from Nano-FMDV_-HS+_ stock. The cells were maintained in 3 ml of medium containing 1% FBS and 100 μl samples of supernatant were taken at 2, 4, 6, 8, 16, and 24 h post infection (hpi) for analysis. The growth kinetics for the Nano-FMDV-_HS+_ was determined by both plaque assay and luciferase assay. Three experimental repeats were performed.

### 2.7 Inhibition of FMDV replication by guanidine hydrochloride

Guanidine hydrochloride (Sigma) treated ZZ-R 127 cells grown in 24-well plates were infected with AvrII-O1K/OUKG-_HS+_ parental virus or Nano-FMDV-_HS+_ at a MOI of 2 by incubation for 1 h at 37° C. Following infection, cells were washed with PBS and MES buffer to remove residual free viral particles and background luciferase, then incubated at 37° C for different periods of time in fresh medium supplemented with 1% FCS and guanidine hydrochloride at a final concentration of 0, 0.004, 0.04, 0.4 or 4 mM. At 7 and 24 hpi, supernatant samples were analysed by luciferase assay and real-time RT-PCR to measure viral yields. Experiments were performed in triplicate.

### 2.8 Virus neutralization test

Micro-neutralisation tests were performed according to the protocol recommended by the World Organisation for Animal Health (Office International des Epizooties (OIE)) (OIE, 2012, Golding et al., 1976). Both AvrII-O1K/OUKG-_HS+_ parental virus and Nano-FMDV-_HS+_ were tested in parallel on IB-RS2 cells against a panel of five sera, comprised of post-vaccination bovine anti-FMDV O1Manisa serum (positive control (Serum 1)), guinea pig anti-FMDV O1Manisa serum (Serum 2), guinea pig anti-FMDV O/Tur5/09 virus like particles (VLP) serum (Serum 3), mouse anti-FMDV O/Tur5/09 serum (Serum 4) and mouse anti-FMDV SAT2 serum (negative control (Serum 5)). Sera were inactivated at 56 °C for 30 min before use. Neat serum stocks were initially diluted 1:8 and then in two-fold dilutions for the tests (1:16, 1:32, 1:64, 1:128, 1:256, 1:512, 1:1024, 1:2048). For each test a 100 TCID50 of virus was used in a total volume of 50 μl. Neutralizing antibody titres, calculated by the Spearmann-Karber method (Kärber, 1931), were expressed as the last dilution of serum that neutralizes 50% of the virus. In addition, the luminescent signal for each sample was determined to investigate if bioilluminesence correlated with the neutralising titres of tested sera.

### 2.9 Viral RNA extraction, conventional and real-time RT-PCR

Viral RNA was extracted using the MagVet™ Universal Isolation Kit (Thermo Fisher Scientific) on a KingFisher^TM^ Flex Robot (Life Technologies). Following RNA extraction, cDNA was generated by using the TaqMan RT reagents Kit (Thermo Fisher Scientific) and hexamer random primers. Platinum HiFi Taq DNA Polymerase (Invitrogen) and the following primer pair were used to amplify the region of viral genome containing the inserted Nluc gene and to provide template for sequencing: forward/5′-CCACTCGGGTGACTGAACTGC-3′ and reverse/5′-TGTCCTCGAGTGATGCCATG-3′.

One-step Callahan 3D quantitative real-time RT-PCR was performed according to the standard protocol of the World Reference Laboratory for FMDV (King et al., 2006).

## 3 Results

### 3.1 Rescue of infectious recombinant FMDVs expressing NLuc

**Fig. 1:**
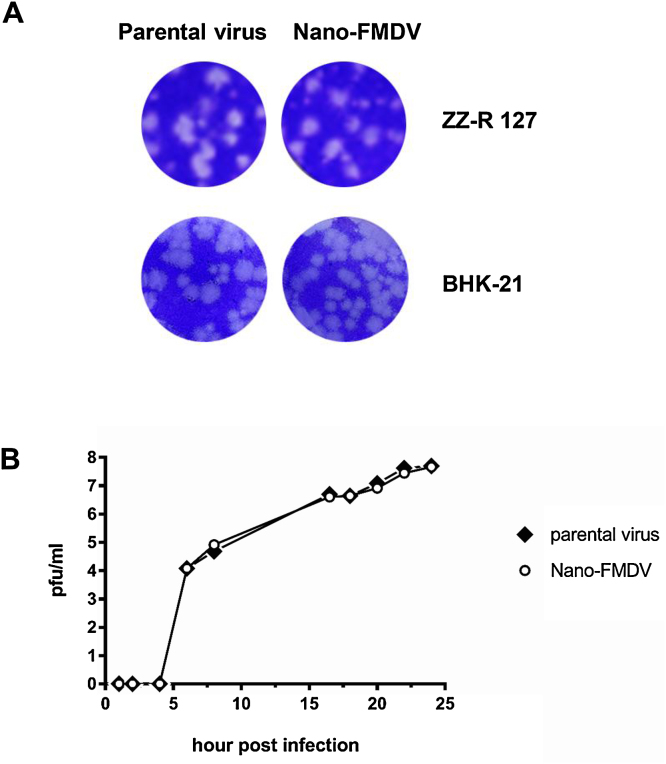
Nano-FMDV-_HS+_ and AvrII-O1K/OUKG-_HS+_ parental virus show similar plaque morphology and growth kinetics. (A) Plaque assays were performed on BHK-21 or ZZ-R127 cells grown in 6-well plates. The plaques were stained at 24 hpi. (B) Growth curve assays were also performed in ZZ-R127 cells with an initial MOI of 0.001. Results are representative of three independent experiments.

In order to generate a novel FMDV that can be used to rapidly monitor viral replication in different cell types, we constructed an infectious copy plasmid encoding a recombinant virus expressing NanoLuc luciferase (Nluc), a small (19.1 kDa), highly stable, bioluminescent protein. To generate this reporter FMDV the NLuc gene was inserted into an existing infectious copy plasmid (herein termed AvrII-O1K/OUKG) previously used to create iLOV FMDV (Seago et al., 2013). This cloning strategy facilitated the successful production of an infectious recombinant FMDV termed Nano-FMDV that expressed NLuc as an N-terminal fusion of the non-structural FMDV 2A protein. BHK-21 cells are the preferred cell line for many laboratories working with FMDV and for vaccine production. Although Nano-FMDV was able to infect the ZZ-R 127 goat cell line that expresses the principal FMDV receptor, integrin αvβ6 (Seago et al., 2012, Burman et al., 2006), it was unable to infect BHK-21 cells which lack αvβ6 (Lawrence et al., 2013) (data not shown). Sequence analysis revealed the inability of Nano-FMDV to infect BHK-21 was likely due to the absence of positively charged amino acids in the capsid, such as VP3 56 Arg that has been shown to facilitate cell entry through interaction with cell surface associated heparan sulphate (Fry et al., 2005, Jackson et al., 1996). A single mutation was therefore introduced into the VP3 region (56 His to Arg) of both the AvrII-O1K/OUKG parental virus and Nano-FMDV to enable the use of heparan sulphate for viral entry and thus infection of BHK-21 cells. Indeed, both viruses, now termed AvrII-O1K/OUKG-_HS+_ and Nano-FMDV-_HS+_ respectively, were able to infect BHK-21 cells, as confirmed by plaque assays (Fig. 1A). In the rest of this paper we describe the use of the O1K/OUKG-_HS+_ parental and Nano-FMDV-_HS+_ viruses.

### 3.2 Nano-FMDV is genetically stable

**Fig. 2:**
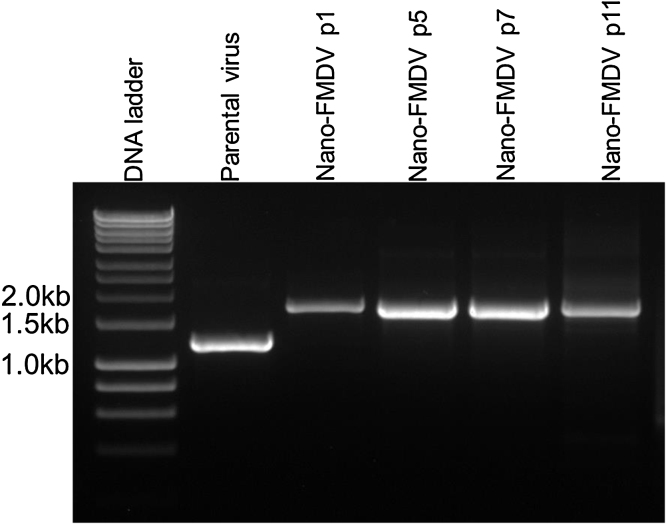
Nano-FMDV-_HS+_ is genetically stable. Nano-FMDV-_HS+_ was serially passaged in BHK-21cells. Viral RNAs from passage (P) 1, 5, 7 and 11 viral stocks were analysed by RT-PCR using a primer pair flanking the insertion site. The AvrII-O1K/OUKG-_HS+_ parental virus was included as a control.

We previously determined that the packaging limit of the FMDV genome for the target insertion site between VP1 and 2A is between 417 nt and 504 nt (Seago et al., 2013). Although insert size is a key factor for the stable retention of exogenous RNA in the FMDV genome, previous research by others on the related picornavirus, poliovirus, has revealed that both the sequence and structure of the inserted RNA may also play a role (Yim et al., 1996, Mattion et al., 1995, Mattion et al., 1994, Lu et al., 1995). With this in mind, stability of the NLuc insert was investigated after serial passage of infectious Nano-FMDV-_HS+_ in ZZ-R 127 cells. After 11 passages in cells, the NLuc ORF was retained intact within the FMDV genome (Fig. 2) with no deletions, as confirmed by sequencing (data not shown).

### 3.3 Nano-FMDV exhibits similar growth kinetics and plaque morphology to the parental virus

Plaque assays were performed on IB-RS2, BHK-21 and ZZ-R127 cells with both the AvrII-O1K/OUKG-_HS+_ parental virus and Nano-FMDV-_HS+_. In comparison to the AvrII-O1K/OUKG-_HS+_ parental virus, Nano-FMDV-_HS+_ exhibited similar levels of infectious viral titres and plaque morphology on all three cell lines (Fig. 1A, only plaques on ZZ-R127 and BHK-21 cells are shown). Next, we assessed the growth of the AvrII-O1K/OUKG-_HS+_ parental and Nano-FMDV-_HS+_ viruses on ZZ-R127 cells. Cells infected (MOI of 1) with either the AvrII-O1K/OUKG-_HS+_ parental virus or Nano-FMDV-_HS+_ exhibited cytopathic effect (CPE) approximately 7 hpi. Therefore, a low starting MOI of 0.001 was used to facilitate comparative increases in infectious virus production over a 24 h period to be made. Virus titres (plaque forming units (PFU)/ml) continued to increase over the 24 h period of infection and no discernible differences in growth kinetics or end point titres were observed between O1K/OUKG-_HS+_ parental and Nano-FMDV-_HS+_ (Fig. 1B).

### 3.4 Luciferase assay can be used to titrate Nano-FMDV

**Fig. 3:**
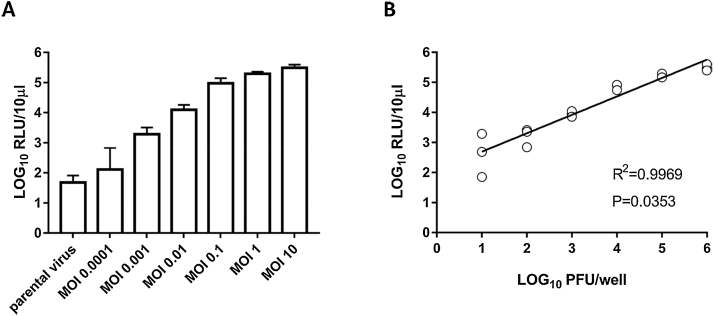
Bioluminescent signal can be used to titrate Nano-FMDV-_HS+_. (A) ZZ-R127 cells were infected for 6 h with AvrII-O1K/OUKG-_HS+_ parental virus at an MOI of 10, or with Nano-FMDV-_HS+_ at the indicated MOI, and light signals were determined for the respective cell culture supernatants. (B) The correlation between virus input and light signal was then analysed using GraphPad Prism 7.0 software. A linear correlation between Log_10_ PFU/well and Log_10_ RLU/10 μl was observed. This experiment was performed in triplicate, and the data is presented as mean ± sd in panel A and individual replicates in panel B.

Plaque and TCID50 assays are commonly used to titrate FMDV. Nano-FMDV-_HS+_ offers the possibility to complement these assays, because levels of luciferase-dependent light emission (light signal) in infected supernatants can be monitored and used as a readout of viral replication. In order to investigate the detection parameters for Nano-FMDV-_HS+_ replication, cells were infected at different MOI (between 0.0001 and 10, equivalent to 10-10^6^ PFU/well) and respective light signals were assessed at 6 hpi. Indeed, light signal above the background could be detected at 6 hpi in supernatant samples of infected cells, and a linear correlation between LOG PFU and LOG RLU was observed (Fig. 3). Hence, these data showed that luciferase assay can be used to monitor Nano-FMDV-_HS+_ replication.

### 3.5 Monitoring Nano-FMDV growth by luciferase assay in the presence of an inhibitor

**Fig. 4:**
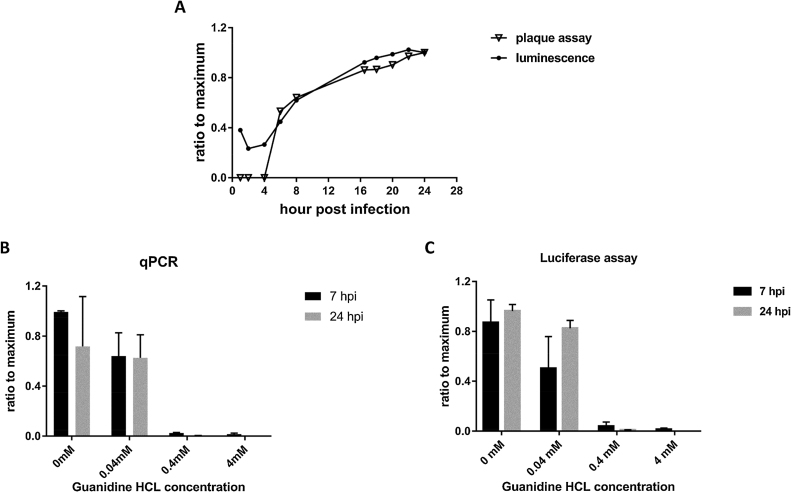
Monitoring FMDV replication by bioluminescence. (A) Two growth curves of Nano-FMDV-_HS+_ generated from the same set of time-course samples, one using plaque assay data (as shown in Fig. 2) and the other luciferase assay data. Both PFU and RLU values were normalized to the respective maximums. (B) Replication of Nano-FMDV-_HS+_ in the presence of guanidine hydrochloride, a known inhibitor of FMDV, at different concentrations was assessed by both quantitative RT-PCR and (C) luciferase assay, and the resulting copy numbers and RLU were normalized to the maximal values obtained in the absence of the inhibitor. Experiments were performed in triplicate and the data are presented as mean ± sd.

To determine if the increase in infectious particles of Nano-FMDV-_HS+_, expressed as PFU/ml in Fig. 2B, was comparable to the increase in bioluminescence signal determined for the same samples, the respective values were plotted alongside each other (Fig. 4A). At each time point the luminescent signal (RLU) and infectivity titre (PFU/ml) were expressed as a ratio to their respective maximum. Between 5 hpi and 24 hpi the curves were nearly identical (Fig. 4A), indicating that the replication of Nano-FMDV-_HS+_ has potential application to monitor the later stages of infection and therefore complement assays that quantify infectious particles.

Next, the potential application of Nano-FMDV-_HS+_ as a unique tool for screening antivirals was examined. To do this, ZZ-R 127 cells were infected with either the AvrII-O1K/OUKG-_HS+_ parental virus or Nano-FMDV-_HS+_ in the presence of a known inhibitor of FMDV, guanidine hydrochloride (Pringle, 1964), at different concentrations (0, 0.04, 0.4 or 4 mM). At 7 and 24 hpi, supernatants were collected and assayed by both Callahan 3D quantitative RT-PCR (Callahan et al., 2002) (Fig. 4B) and luciferase assay (Fig. 4C); respective copy numbers and RLU were normalized to the values obtained in the absence of the inhibitor. Almost complete inhibition of Nano-FMDV-_HS+_ replication, as determined by both RT-PCR and luciferase assay, was observed at 7 and 24 hpi in the presence of 0.4 and 4 mM guanidine hydrochloride. As expected, the lower concentration of 0.04 mM guanidine hydrochloride resulted in an observable but insignificant inhibition as determined by both RT-PCR and luciferase assay (p > 0.05). More importantly the inhibition measured at 7 and 24 hpi for all concentrations of the inhibitor were comparable (p > 0.05). The above results suggest that Nano-FMDV-_HS+_ holds potential for the development of cost-effective, rapid methods that facilitate high-throughput analysis of viral replication and antiviral screening.

### 3.6 Virus neutralisation test using Nano-FMDV

**Fig. 5:**
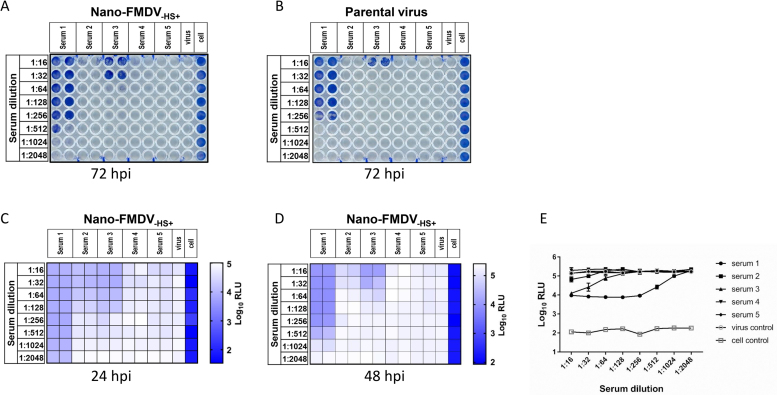
Virus neutralisation test using Nano-FMDV-_HS+_ and bioluminescence. Nano-FMDV-_HS+_ (A) and AvrII-O1K/OUKG-_HS+_ parental virus (B) were tested against a panel of 5 sera (see material and methods section) using the conventional virus neutralisation test assay. Luciferase assays were performed using 10 μl of supernatant taken from the Nano-FMDV-_HS+_ plate (shown in A) at 24 hpi and 48 hpi and the bioluminescent results were presented as heat maps (C and D, respectively). (E) The 48 hpi bioluminescent results for Nano-FMDV-_HS+_ presented as mean ± sd. Experiments were performed in duplicate.

The virus neutralisation test is an OIE prescribed test for international trade (OIE, 2012). It is sensitive and serotype specific, and plays an important role in FMDV diagnosis and vaccine matching. The current protocol recommended by OIE is time consuming and labour intensive. A more efficient process would be advantageous. One possible approach to achieve this goal is the use of reporter viruses expressing luciferase (Zhou et al., 2013, Fuentes et al., 2013, Rostad et al., 2016, Zhang et al., 2016a, Karlsson et al., 2015). Therefore, we next investigated if Nano-FMDV-_HS+_ could be used to rapidly monitor the presence of neutralising antibodies. Initial experiments compared the AvrII-O1K/OUKG-_HS+_ parental virus and Nano-FMDV-_HS+_ in a conventional neutralisation test against five sera. At 72 hpi, the neutralisation titres of serum 1 were 1:355 and 1:512 against the parental virus and Nano-FMDV-_HS+_, respectively, and the respective tires for serum 3 were 1:22 and 1:45 (Fig. 5). No protection was detected at the dilution of 1:16 and above for sera 2, 4 and 6 against both viruses. We then analysed cell culture supernatants taken from the Nano-FMDV-_HS+_ plate at 24 hpi and 48 hpi by luciferase assay and prepared heat maps to assess the data. The neutralisation pattern revealed by the conventional method (Fig. 5A) could be reproduced at 48 hpi using bioluminescence signal (Fig. 5D). Indeed, even at 24 hpi a similar neutralisation pattern was observed (Fig. 5C). These results suggests the possibility of developing a rapid and high-throughput method to detect neutralising antibodies against FMDV.

## 4 Discussion

Bioluminescence has been used in the study of many other viruses *in vivo* and *in vitro*. In these studies, accommodation of the inserted marker sequence and the generation of infectious virions has been successful (Coleman and McGregor, 2015, Hutchens and Luker, 2007). However, due to the size restraints imposed by the packaging limits of the FMDV capsid, there has been no report of a viable FMDV in infected cell culture supernatants expressing a luciferase reporter. Following our success of making a viable FMDV expressing a fluorescent reporter, iLOV-FMDV, we now report for the first time the successful generation of an infectious luciferase reporter FMDV (Seago et al., 2013). In this study, we took advantage of the newly developed Nanoluciferase by Promega, NanoLuc^®^ (Nluc), which is a small (19.1 kDa) engineered luminescent reporter. Developed as a reporter protein for human cell lines (Hall et al., 2012b), Nluc has also been shown to function in bacteria (Loh and Proft, 2014) and yeast (Masser et al., 2016). Originating from the deep sea shrimp, *Oplophorus gracilirostris* (Inouye et al., 2000), NanoLuc (Nluc) is a novel engineered luciferase that is about 150-fold brighter than either firefly (*Photinus pyralis*) or *Renilla reniformis* luciferase, and utilises a novel optimised substrate, a coelenterazine analog (furimazine), to produce high intensity, glow-type luminescence. The luminescent reaction is ATP-independent and designed to suppress background luminescence for maximal assay sensitivity (England et al., 2016). Due to these advantageous features, NLuc has been quickly applied in the development of new tools for antibody screening (Boute et al., 2016) and to characterise protein-protein interactions (Mo and Fu, 2016, Zhao et al., 2016), image tumours (Germain-Genevois et al., 2016, Schaub et al., 2015, Kamkaew et al., 2016), and study pathogens, including viruses (Karlsson et al., 2015, Anindita et al., 2016, Nishitsuji et al., 2015, Seay et al., 2015, Sun et al., 2014, Tran et al., 2013), batcteria (Loh and Proft, 2014, Zhang et al., 2016b) and parasites (De Niz et al., 2016). Recently Karlsson et al. have successfully performed a bioluminescence imaging study on ferrets infected by a NLuc tagged Influenza A virus, and have identified infections in animals that would have otherwise been missed by traditional methods (Tran et al., 2013).

Previously, we used progressively larger insertions of GFP open reading frame (ORF) to show insertions up to 417 nt in length at the VP1/2A site are stably retained in the FMDV genome (Seago et al., 2013). However, insertions ≥ 504 nt were unstable and excised, through a yet undefined mechanism, to yield infectious virions. The cut-off point between these differently sized insertions was not investigated. Interestingly, similar packaging limitations of approximately 400 nt have been reported for poliovirus, a related picornavirus (Mattion et al., 1995, Mattion et al., 1994, Lu et al., 1995, Mueller and Wimmer, 1998). These packaging limitations explained our failure to generate a *Renilla* luciferase tagged FMDV. In contrast, the Nluc RNA stably incorporated into the parental viral genome without any significant effects on viral growth or plaque phenotype. As the Nluc ORF is marginally larger (513 nt) this suggests that the packaging capacity of the FMDV genome may have slight plasticity. However, it must be noted that in addition to the size of exogenous RNA inserted into the FMDV genome, other factors have been shown to influence the stability of insertions into the poliovirus genome, including the site of insertion, the sequence and its respective structure (Yim et al., 1996, Mattion et al., 1995, Mattion et al., 1994, Lu et al., 1995).

We have demonstrated here that the bioluminescence produced following infection with Nano-FMDV-_HS+_ can be used to monitor viral replication. In our study, comparison of infectious particle production and bioluminescence signal of parental and Nano-FMDV-_HS+_ viruses showed good correlation between 5 hpi and 24 hpi. The comparatively higher values observed for bioluminescence prior to 5 hpi may have been due to the use of unpurified virus samples and the presence of background levels of NLuc protein. Nevertheless, the use of bioluminescence at later stages of FMDV replication still has application for the high-throughput screening of antivirals and antibodies. Nano-FMDV-_HS+_ can be used to complement conventional neutralisation assays by facilitating automated longitudinal data collection. In addition, Nano-FMDV-_HS+_ has a potential use in studies investigating viral pathogenesis, cell tropism, small animal models and vaccine efficacy. Current FMD vaccines are comprised of chemically inactivated whole virus preparations. In order to produce high virus titres prior to the inactivation process, vaccine manufacturers use BHK-derived suspension cell lines that can rapidly grow to high densities. New seed stock strains must first be cell adapted to facilitate infection of such cell types and cell stocks need to be routinely monitored to ensure they support consistent levels of virus replication. Nano-FMDV-_HS+_ offers a new tool to study cell adaption, to monitor viral replication for quality assurance purposes, to screen for new cell lines that support FMDV growth, and to identify conditions that support optimal viral replication. In addition, by facilitating plate-based quantitative assays, Nano-FMDV-_HS+_ complements our previously developed reporter FMDV, iLov FMDV, which has particular application for monitoring FMDV infection *in vitro* in real-time (Seago et al., 2013). Future work will investigate application of Nano-FMDV-_HS+_ in the context of both *in vitro* and *in vivo* infection.

## Conflict of interest statement

The authors do not have any conflict of interest to declare.
